# Thermal Stability and Melting Dynamics of Bimetallic Au@Pt@Au Core-Shell Nanoparticles

**DOI:** 10.3390/s23125478

**Published:** 2023-06-10

**Authors:** Vadym Borysiuk, Iakov A. Lyashenko, Valentin L. Popov

**Affiliations:** 1 Department of System Dynamics and Friction Physics, Institute of Mechanics, Technische Universität Berlin, 10623 Berlin, Germany; v.borysiuk@campus.tu-berlin.de (V.B.); v.popov@tu-berlin.de (V.L.P.); 2 Department of Nanoelectronics and Surface Modification, Faculty of Electronics and Information Technology, Sumy State University, 40007 Sumy, Ukraine; 3 Department of Applied Mathematics and Complex Systems Modeling, Faculty of Electronics and Information Technology, Sumy State University, 40007 Sumy, Ukraine

**Keywords:** bimetallic nanoparticles, core-shell structure, melting, molecular dynamics, Lindemann index

## Abstract

Thermal stability is an important feature of the materials used as components and parts of sensors and other devices of nanoelectronics. Here we report the results of the computational study of the thermal stability of the triple layered Au@Pt@Au core-shell nanoparticles, which are promising materials for H_2_O_2_ bi-directional sensing. A distinct feature of the considered sample is the raspberry-like shape, due to the presence of Au nanoprotuberances on its surface. The thermal stability and melting of the samples were studied within classical molecular dynamics simulations. Interatomic forces were computed within the embedded atom method. To investigate the thermal properties of Au@Pt@Au nanoparticles, structural parameters such as Lindemann indexes, radial distribution functions, linear distributions of concentration, and atomistic configurations were calculated. As the performed simulations showed, the raspberry-like structure of the nanoparticle was preserved up to approximately 600 K, while the general core-shell structure was maintained up to approximately 900 K. At higher temperatures, the destruction of the initial fcc crystal structure and core-shell composition was observed for both considered samples. As Au@Pt@Au nanoparticles demonstrated high sensing performance due to their unique structure, the obtained results may be useful for the further design and fabrication of the nanoelectronic devices that are required to work within a certain range of temperatures.

## 1. Introduction

Metallic nanoparticles (NP) are of great interest for various fields of nanotechnology, as they may provide new possibilities for designing and fabricating alternative material structures with characteristics that are not observed in original bulk samples [1,2,3]. The increased popularity of bimetallic nanostructures is caused by their better electronic, chemical, and optical properties compared to monometallic ones [4,5].

Modern methods of synthesis allow to obtain NPs of different chemical composition and geometrical shapes. Based on the mixing scheme, bimetallic nanoparticles can be divided into three main types [1,6]: core-shell structures, heterostructures, and solid solutions. Various combinations of noble metals with core@shell structure, where the inner core is surrounded by one or several shells of another material in the form of concentric spherical and cubic particles and nanowires, are among the most popular variants of the chemical composition and structure of bimetallic NPs [1,2,3,7,8,9]. In particular, the presence of an additional metal is a promising modification in the field of nanocatalysis, as such structures allow to minimize the use of expensive materials while at the same time maximizing the area of catalytic surface and increasing the catalytic properties compared to monometallic nanoparticles even at lower temperatures [5,9]. Therefore, bimetallic alloys are actively used for more effective interactions of the active centers for homogeneous [10], heterogeneous [11,12,13], photo [14,15] and electro catalysts [16,17].

Core@shell nanoparticles have numerous applications in nanoelectronics [2], and in particular in various sensing technologies, namely, in drug delivery biosensing [18], multifunctional sensor systems [19], gas sensors [20,21,22], and chemical sensors for hydrogen peroxide, glucose, formaldehyde [23], ethanol [24], and others [19]. In addition to classic bimetallic core@shell NPs with a single core covered with one layered shell of other metal, triple-layered structures are also implemented for sensing [25,26,27,28,29].

The study of the structural properties of the surface of metal nanoparticles at the atomistic level is of great importance for their synthesis and applications, since the surface structure mainly determines their chemical and physical properties. At the same time, thermal stability is a crucial attribute of nanoparticles as they must remain in solid state to function as a part of nanoelectronic devices, sensors and other applications. In this regard, the melting of metal particles has been intensively studied both experimentally and theoretically during the last decades [30,31].

It is worth to note that melting, as a transition from the solid to the liquid phase, of metal nanoparticles begins mainly at the surface and gradually spreads to the center [32]. However, the recent studies also revealed the phenomenon of the dependence of the melting temperature of nanostructures on their size, which was not observed in bulk materials. Thus, for NPs with a size of a several nanometers, the reduction of the melting temperature may be tens or even hundreds of degrees compared to bulk samples of the same material [32,33]. This phenomenon is called melting point depression [32]. Melting point depression is most evident in nanowires, nanotubes, and nanoparticles, which all melt at lower temperatures than the bulk structures of the same material. Disagreements in the melting point occur due to the fact that nanoscaled materials have a much larger surface-to-volume ratio than bulk materials, which dramatically changes their thermodynamic and thermal properties. Bimetallic nanoparticles with core@shell structure show even greater differences in thermodynamic properties compared to monometallic nanostructures due to the different values of melting points between the two elements. Thus, the melting of core@shell NPs usually occurs in a temperature range close to the melting temperature of the shell material, especially for NPs with small core sizes. However, depending on the size, geometrical shape, and chemical composition of certain nanoparticles, various scenarios of melting dynamics are possible.

The study of the thermal stability of core@shell nanoparticles solely from experimental data is a hard challenge due to the small scale and complexity of their structure. Moreover, thermal properties also may vary for particular NP samples of the same composition and structure. Thus, it is also necessary to use analytical models and computer simulations of physical processes that occur in nanomaterials at the atomistic level during heating and melting. Therefore, the melting of nanoparticles is intensively studied using molecular dynamics (MD) simulations (see for example [34,35,36,37]). The main method of studying the thermodynamic parameters of nanoparticles using MD technique is the analysis of temperature dependences of the potential energy of samples, as well as the calculation of other structural parameters, such as the Lindemann index [38] and radial distribution functions (RDF) [39].

In this paper we report the results of the atomistic simulation of the melting dynamics of Au@Pt@Au core-shell nanoparticle with a structure similar to the NPs reported in [29]. In particular, the authors report on the synthesis of bimetallic Au@Pt@Au core-shell nanoparticles on graphene oxide nanosheets, with Au core, Pt inner shell, and outer Au protuberances. Fabricated nanocomposites showed a high efficiency for H_2_O_2_ bi-directional amperometric sensing due to their unique physical properties and structure with exposed surfaces of two different metals [29]. Thus, studying the thermal stability of these NPs, and determining the range of temperatures within which the samples preserve their unique structure, is an important task for nanoelectronics and sensing technologies. Results obtained in our study as well as the described melting dynamic of the NP may be useful for further development and tuning of the sensing technology proposed in [29].

## 2. Simulation Setup

As it was mentioned in the introduction, we performed an atomistic modeling of bimetallic triple layered Au@Pt@Au nanoparticle with the structure and chemical composition similar to samples synthesized in [29]. A distinct feature of these NPs is a raspberry-like shape due to the outer shell being composed of Au protuberances grown on an inner Pt layer. The authors reported [29] that such a structure showed higher electrocatalytic performance compared to triple layered Au@Pt@Au NPs with a smooth outer gold shell. Therefore, studying the thermal stability of the mentioned raspberry-like shaped nanoparticle is the main purpose of the performed simulations.

**Figure 1:**
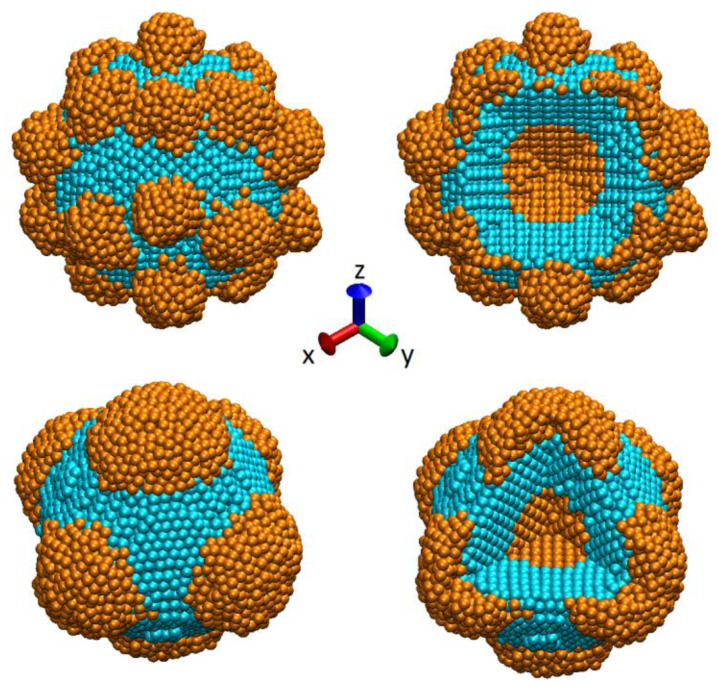
Example of initial atomistic configurations of the studied NP with small (top panel) and large (bottom panel) Au protuberances (general (left) and cross-segmental (right) views). All snapshots were prepared using the visual molecular dynamics (VMD) software, version 1.9.1 [40].

**Figure 2:**
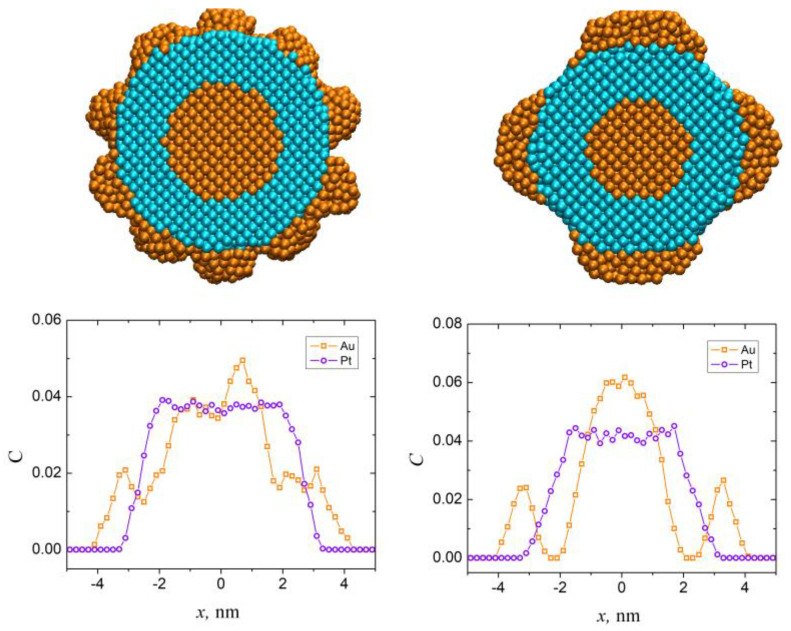
Cross-sectional views and linear distributions of the concentrations of Au and Pt atoms of the sample 1 (top left and bottom left, respectively) and sample 2 (top right and bottom right, respectively).

An initial configuration of the studied nanoparticle was obtained by assigning Cartesian coordinates to Au and Pt atoms according to their ideal crystal lattice positions. First, the Au core and Pt inner shell of spherical shapes were prepared, after which uniformly distributed Au nanoprotuberances close to a hemispherical shape were added onto the surface of the Pt shell. In the initial configuration of the sample, Au nanoprotuberances were positioned at approximately the same distance from each other. The average diameter of nanoparticles synthesized in [29] is reported to be 24.6 nm; however, to reduce computational time, we consider approximately three times smaller model samples with a diameter *d*~8.0 nm. It is worth noting that while we aimed to study the thermal stability of the NP of this particular structure, real samples of Au@Pt@Au nanoparticles synthesized in [29] have different sizes and shapes of the outer raspberry-like Au layers; thus, we consider two samples with different initial configurations. The atomistic configuration of the studied NPs at the beginning of simulations are shown in Figure 1 (compare to related images in [29]). As it can be seen from the figure, the main difference between the considered model samples is the number and size of the outer Au nanoprotuberances. Thus, the top panel of Figure 1 shows the sample with a large amount of small-sized Au nanoprotuberances, while the bottom panel of the figure presents the NP with several large Au nanoprotuberances at the outer shell. Here and below we will refer to these samples as sample 1 and sample 2, respectively. The total amount of atoms is 12,416 and 10,318 in sample 1 and sample 2. Each sample consists of approximately 65% of Pt atoms and 35% of Au atoms. At the same time sample 1 has a larger Au core, compared to sample 2, as can be seen from the cross-sections of the samples shown in Figure 2.

Figure 2 also shows the linear distributions of the concentrations of both Au and Pt atoms. The distribution of the concentration of the certain atoms can be considered a direct qualitative measure of the triple layered structure of the Au@Pt@Au nanoparticle. Thus, with the aim to investigate the thermal stability further, we will compare these dependencies, calculated at different temperatures, together with other structural parameters, such as the Lindemann index and radial distribution functions.

In our study, we adopted the classical molecular dynamics technique, where the interaction between atoms are described within the widely used embedded atom method (EAM). A full description of the EAM approach can be found in [41]. Interatomic forces, corresponding coordinates, and velocities of the atoms were calculated using the previously developed in-house code for parallel computing on GPU, which was successfully employed to study the thermal stability of various nanomaterials [42,43]. Simulations were performed in ideal vacuum and free boundary conditions. The temperature of the simulated samples was gradually increased from the initial value of 300 up to 2250 K with an increment of 50 K through a Berendsen thermostat [44] according to the algorithm described in [42] by rescaling the corresponding atomic velocities. Every 50 K samples were maintained at a given temperature for 2 × 10^5^ time steps. During that time data needed for the structural analysis of the sample were recorded and the simulation continued. All simulations were carried out on the desktop system based on NVIDIA Tesla P100 16 GB PCI-E GPU with CPU Intel Core i5-8600K (with 4.6 GHz frequency). An analysis of the data, recorded throughout the whole simulation period, is given in the next section.

## 3. Results and Discussion

The thermal stability of the nanoparticles from molecular dynamics simulations is typically studied by analyzing the temperature dependencies of both the Lindemann index and potential energy. The Lindemann index is commonly used in MD simulations parameter that indicates changes in the crystal structure of the sample. Generally, a local Lindemann index *q_i_* can be calculated separately for every atom *i* as [38]:

(1) $$q_{i}=\frac{1}{N-1}\sum_{i\neq j}\frac{\sqrt{\langle r_{ij}^{2}\rangle -{\langle r_{ij}\rangle }^{2}}}{\langle r_{ij}\rangle }$$

where *n* is a total number of atoms in the system, *r_ij_* is the distance between two atoms *i* and *j*, and angle brackets denote the time averaging at a given temperature. To track changes in the crystal structure of the sample, usually the temperature dependence of the Lindemann index of the whole sample *Q* as an average over all atoms is used. Thus, a temperature at which the Lindemann index *Q* reaches its critical value *Q_c_* is considered an indicator of the melting point of the sample. In general, values of *Q_c_* in the range 0.1 ÷ 015 is used as a melting criterion for bulk samples. However, for nanomaterials, significantly lower values of *Q_c_* ≈ 0.03 is commonly used in MD studies [38,42]. Moreover, as it is reported in the literature, the melting of the Pt-Au nanoparticles with a core-shell structure characterized by the *Q_c_* ≈ 0.03 ÷ 0.2 range of values [35], at the same time, for gold nanostructures values *Q_c_* ≈ 0.02 ÷ 0.07 are reported as a melting criterion [45]. Besides the actual value of *Q_c_*, temperature dependence *Q*(*T*) also provides information about structural changes in the sample due to melting. Thus, it is known that the Lindemann index exhibits small values at low temperatures and slowly increases with the temperature growth. As the melting of the sample begins *Q*(*T*) demonstrates fast nonlinear growth. After a sample is melted, the growth of Lindemann indexes is expected to slow down with further increasing of the temperature. It is also worth to note, that potential energy of NP exhibits similar behavior during heating, with the regions of slow and fast growth around the phase transition point. Such type of behavior is typically observed in MD simulations of the thermal stability of nanoparticles and other nanomaterials [34,35,38] and therefore, examining the temperature dependencies of both Lindemann indexes and potential energy, as well as estimated the critical value *Q_c_* one can obtain in the picture of structural changes in the studied sample.

**Figure 3:**
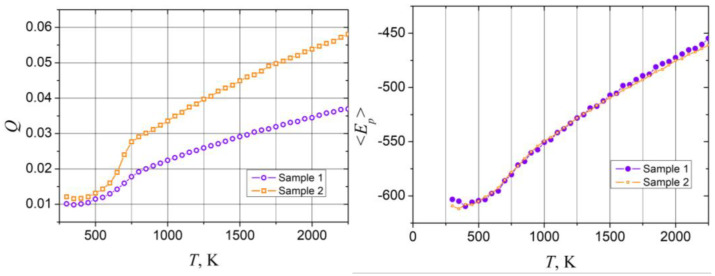
Temperature dependencies of the average Lindemann index (left panel) and potential energy (right panel) of sample 1 and sample 2 (curves denoted in the figure).

**Figure 4:**
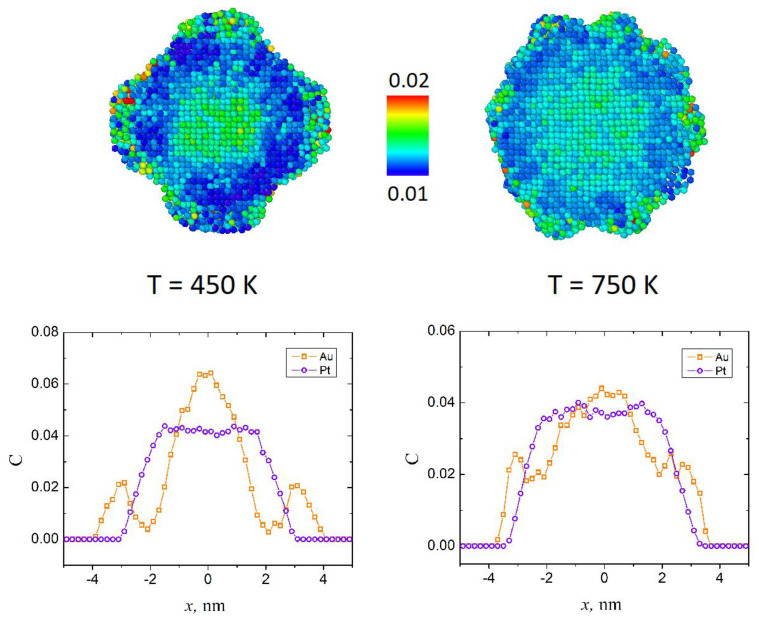
Distribution of the atoms according to the magnitude of Lindemann index within the volume of the sample at different temperatures for sample 2 (top left) and sample 1 (top right) and related linear distributions of the concentrations of Au and Pt atoms for sample 2 (bottom left) and sample 1 (bottom right).

Calculated temperature dependencies of Lindemann index and potential energy for sample 1 and sample 2 are shown in Figure 3. As the figure shows, the presented dependencies *Q*(*T*) and <*E_p_*>(*T*) have typical form where the fast growth of the Lindemann index indicating the start of the melting process begins around *T* = 600 K for both samples. Besides this, dependencies <*E_p_*>*(T)* calculated for both samples have close values, while the Lindeman index of the sample 2 exhibits significantly larger values, comparing to sample 1 after the beginning of melting (in the *T* > 600 K region). Moreover, *Q*(*T*) calculated for sample 2 reaches a critical value 0.03 at approximately 800 K, while for sample 1 the same magnitude is reached at a significantly higher temperature (approximately 1500 K). At the same time, the slowdown of the Lindemann index growth is observed around 800 K for both samples. Notably for sample 2, this temperature point coincides with critical value *Q_c_* ≈ 0.03, while for sample 1 it is characterized by a smaller magnitude *Q* ≈ 0.02. The mentioned peculiarity may be caused by the different shape of the outer layer of the samples as well as by the different numbers of atoms involved in simulations for both cases. Thus, the Lindemann index as the measure of the thermal disorder indicates the average amplitude of the thermal fluctuations of atoms, i.e., atoms with a large average amplitude of motion due to the temperature are characterized by a larger Lindemann index and vice versa. Therefore, different amounts of surface atoms, which typically have large movability, may cause the difference in the magnitude of Lindemann index. Examples of the distribution of the atoms according to their Lindemann index in the volume of sample 1 and sample 2 together with linear distributions of Au and Pt atom concentrations are presented in Figure 4.

The top left panel of the figure shows the cross-sectional view of the sample 2 at 450 K, before melting. At this temperature, three regions with a different range of values of the Lindeman index are noticeable within the sample volume, namely, central part of the sample, related to the Au core consisting of atoms with a relatively high *q_i_* compared to the adjacent region, related to Pt inner shell, which contains atoms with a lower *q_i_*. The third region related to surface atoms consists of Au nanoprotuberances as well as a certain amount of surface atoms from Pt inner shell, is characterized by the highest Lindemann indexes. This tendence is also clearly visible in the top right panel of the figure, which presents sample 1 at 750 K. Such type of behavior is qualitatively correct, as platinum has a significantly higher melting point compared to gold (2041 and 1337 K, respectively) and thus the Pt shell is expected to melt at higher temperatures, and therefore Pt atoms are characterized by smaller magnitudes of the Lindemann index, compared to Au atoms. At the same time, both Au and Pt atoms on the surface of NP tend to have higher *q_i_* values than the atoms within the sample. As it can be seen from the related linear distributions of Au and Pt concentrations at specified temperatures (bottom panels of the Figure 4), the triple layered core-shell structure is preserved within both samples.

**Figure 5:**
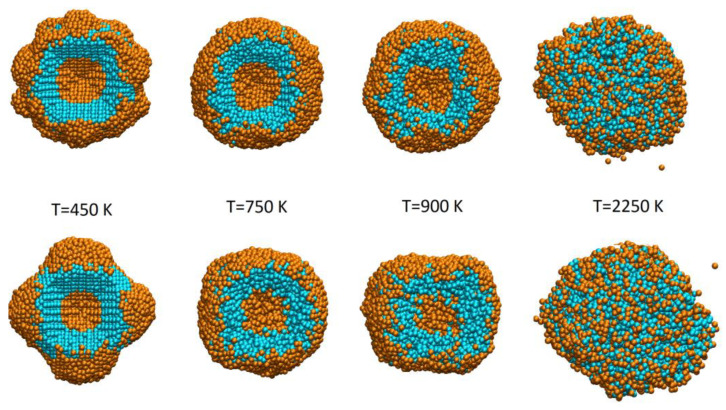
Snapshots of atomistic configurations of the samples at different temperatures. Top panel shows cross-segmental views of the sample 1, while bottom panel represents the same for sample 2.

MD simulations allow us to visualize the melting process of the model samples. Complete processes of the melting of both NPs (general view and cross section) are presented in Supplementary Materials, Video S1. Selected snapshots of atomistic configurations at different stages of melting are also shown in Figure 5.

As it can be seen from the snapshots and corresponding Supplementary Video S1, the dynamics of the thermal degradation of the crystal structure of both samples is similar. At small temperatures (approximately below 600 K, as it follows from the dependencies shown in Figure 3), both samples preserve their fcc crystal structure, triple layered core-shell composition, and raspberry-like surface. Significant changes in the structure of NP occur after the melting begins, (approximately above 600 K according to the temperature dependence of the Lindemann index). As it follows from the presented snapshots, nanoprotuberances at the surface of samples rearrange into an almost smooth outer Au shell. However, within the sample, atoms preserve their fcc lattice and core-shell structure. With further temperature growth, the long-range ordering of the crystal structure is destroyed, and samples no longer maintain the Au@Pt@Au triple layered core-shell structure.

**Figure 6:**
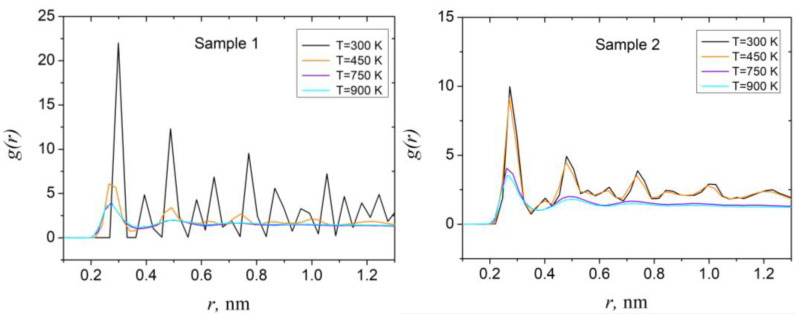
RDF calculated for sample 1 (left panel) and sample 2 (right panel) at different temperatures (temperature values denoted in the figure).

To obtain the quantitative characteristic of the crystal structure of NPs, radial distribution functions were computed from atomistic configurations of samples at different temperatures using common expression [39]:

(2) $$g(r_{n})=\frac{Vh_{n}}{2\pi N^{2}r_{n}^{2}\Delta r}$$

where *h_n_* is the number of atom pairs (*ij*) for which distance between *i* and *j* satisfies the condition (*n* – 1)Δ*r* ≤ *r_ij_* ≤ *n*Δ*r* (if Δ*r* is rather small), *V* is the volume of the sample, *N* is, as above, total number of atoms, *r_n_* = (*n* – 1/2)Δ*r*. Obtained dependencies are shown in Figure 6. As the figure shows, the RDF calculated for both samples have a shape typical for crystals, with peaks related to interatomic distances between the atoms, arranged in a crystal lattice. With the temperature growth, the height of the peaks decreases and long-range ordering is no longer observable. This situation is also noticeable in related snapshots of atomistic configurations, shown in Figure 5. RDF calculated at higher temperatures are not convenient for comparison, due to significant changes of the samples’ volume and partial evaporation of the surface atoms; therefore, these dependencies will not be shown here.

**Figure 7:**
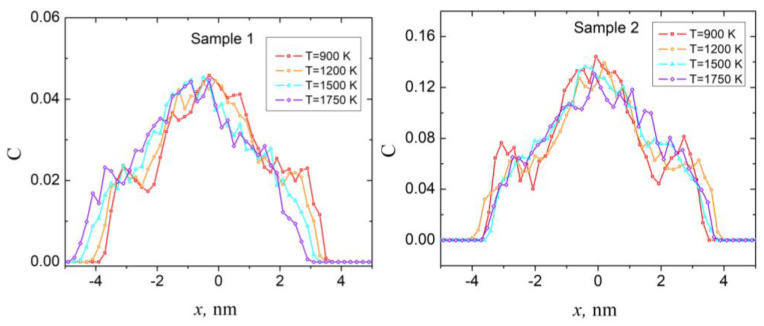
Linear distributions of the concentrations of Au atoms for sample 1 (left panel) and sample 2 (right panel) at different temperatures (temperature values denoted in the figure).

Finally, to obtain qualitative characteristics of the core-shell structure of NP, we calculated the linear distributions of the concentrations of Au atoms at different temperatures. As it follows from Figure 2 and Figure 4, the distribution of concentrations of Au atoms within the samples is characterized by lateral minima and maxima, related to the presence of the Pt inner shell; therefore, these dependencies are convenient for tracking the changes in NP compositions. Obtained results are presented in Figure 7.

As the left panel of Figure 7 shows, at temperatures below 1200 K, peaks on the distribution of Au atom concentration related to outer Au shell (around ±3 nm) are still clearly noticeable, which indicates that core-shell structure also preserved within the sample. At temperatures above 1200 K, NP no longer maintains its core-shell structure, as the Au atoms are almost uniformly distributed within the volume of the sample. At this temperature samples have a structure similar to the Au-Pt alloy in an amorphous state; however, layered structure may be locally preserved in certain parts of the sample. At the same time, according to dependencies shown in the right panel of Figure 7, within sample 2 the destruction of the core-shell structure is already observed at 1200 K.

As described in the literature, besides the well-known melting point depression [32], the size of the core@shell sample may also affect the thermal stability of NP as the changing of the radius of nanoparticle leads to the related changes in the concentration of core and shell atoms, surface to volume ratio, relative core and shell thickness, etc. Thus, for example, for Au@Pt core@shell nanoparticles, with an increasing NP radius at a fixed radius of the core, the melting point also increases, while at fixed shell thickness, it decreases with NP radius growth [46,47]. The behavior of triple layered Au@Pt@Au nanoparticle can be even more complex, due to the presence of additional shell. Therefore, additional studies on this matter are required. However, tracking the size dependence of the melting point of the raspberry-like shaped NP within a small range (several nanometers) of its radius is a hard challenge, as for each sample of a different radius the relative size of nanoprotuberances at its surface and therefore area of free surface will also be different, which may affect the melting behavior as well.

To investigate how the size of the sample affects its thermal stability, we performed additional simulations for five different samples of the Au@Pt@Au nanoparticle with smooth surface. Also, as it was mentioned above, the melting point of the NP depends on relative sizes of core and shell, as well as on the concentrations of Au and Pt atoms [46,47]; thus, to limit the factors, affecting the NP melting, simulations of NP with different sizes were performed at a fixed size of Au core of about 1 nm, while the relative concentrations of Au atoms in NP were kept approximately 70%. The total number of particles involved in simulations varied from 1966 to 15,828, with a radius from 2.0 to 4.0 nm.

**Figure 8:**
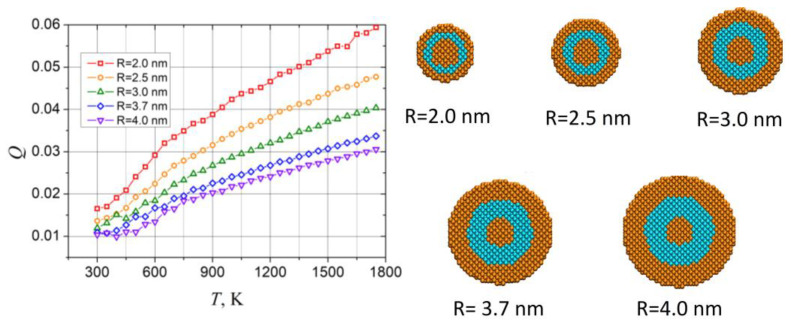
Temperature dependencies of the average Lindemann index for five samples of a different radius (left panel), and initial atomistic configurations of the studied samples (right panel). Sizes of the samples are denoted in the figure.

Temperature dependencies of Lindemann indexes calculated for all five studied samples together with their initial configurations are shown in Figure 8, while changes of atomistic configurations during heating are presented in Supplementary Video S2. As can be seen from the figure, with the growth of the NP radius, related *Q*(*T*) curve reaches critical value *Q_c_* = 0.03 at higher temperatures, which indicates a corresponding shift of the melting point. Moreover, as it follows from the atomistic configurations from the Supplementary Video S2, a disruption of the initial structure was observed at lower temperatures for smaller samples.

It is worth noting that real Au@Pt@Au samples may show a different behavior than was obtained in the simulations for several reasons. For example, real samples have larger sizes and a slightly different structure that is hard to exactly reproduce within the proposed approach. Moreover, our simulations were performed in ideal vacuum conditions whereas real samples are synthesized in a certain chemical solution, and may be exposed to air or other environments during the experiments and therefore oxides and various chemically active groups may be present at the surfaces of NPs, which may dramatically change their properties.

To the best of our knowledge, an experimental study of heating and melting of the Au@Pt@Au nanoparticles has not been reported in the literature for now, while experimental investigations of the thermal stability of bimetallic core-shell nanoparticles of other chemical compositions and at different conditions are described (see for example [48,49,50]). However, most of the experimental studies concerning the heating of core-shell NPs are focused on their catalytic performance, oxidation and chemical reactivity. Nevertheless, our study qualitatively reproduces the general behavior of the core-shell NP during heating, where the destruction of the core-shell structure of bimetallic nanoparticle and formation of the mixed alloy of two metals are observed prior to melting. For example, such type of behavior was reported for Ni-Co core-shell NPs [48]. Namely, in the experiments, the disrupted core-shell structure was observed above 440 °C.

While experiments regarding the thermal stability of Au@Pt@Au core-shell nanoparticles have not been reported yet, a number of MD simulations of the melting of Au-Pt core shell nanoparticles of different sizes and several variants of core-shell compositions are available [35,46,47,51]. Thus, according to the data reported in [51] among the samples with either Au or Pt core and single shell, NPs with Pt-core and Au-shell are more stable comparing to samples with Au-core and Pt-shell while simulations of the thermal stability of samples with an icosahedral structure, Pt core, Au shell and different concentrations of Pt and Au atoms revealed the increase of the melting temperature with the growth of concentrations of Pt atoms [35]. Moreover, as it follows from [47], the thermal and structural stability of Au@Pt nanoparticles can be improved by decreasing the size of the Au core and increasing the thickness of the Pt shell. Besides this, the authors report the inhomogeneous melting for NPs with moderate or large Au core [47]. At the same time, according to the results of simulations of the melting of Au@Pt core@shell nanoparticles performed in [46], core size affects the melting temperature in two regions. Namely, small core almost has no effect on the melting temperature of NP which in this case is determined by Pt shell melting, while for a large core, with a radius larger than critical, the melting temperature linearly decreased with the growth of the core radius.

Summarizing, we believe that the obtained results concerning the thermal stability of Au@Pt@Au nanoparticles may become useful in further research within the specified topic, as how original research [29] reports, the raspberry-like surface of the NP is crucial for excellent sensing performance, compared to the smooth outer Au shell. Therefore, it is important to know the range of temperatures within which the specific structure of NP is preserved. Furthermore, Au@Pt@Au nanoparticles with a smooth outer shell can also be used for sensing purpose; thus, the range of temperatures where NP maintains its general core-shell structure is also of significant interest.

## 4. Conclusions

We reported the classical molecular dynamics study of the thermal stability of several different samples of Au@Pt@Au nanoparticles. The performed simulations allowed us to obtain an atomistic configuration and structural parameters of the samples. From the obtained data we can conclude that the thermal degradation of the studied samples with Au nanoprotuberances develops in two stages. First, the Au nanoprotuberances at the surface of NP that forms its raspberry-like shape rearranges into a smooth Au outer shell at approximately 600 K. Next, at higher temperatures, the destruction of the triple layer core-shell structure of the sample is observed and NP is no longer solid. At the same time, a shift of the melting point to higher temperatures with the growth of thr NP diameter was observed for the Au@Pt@Au samples with a smooth surface.

## Data Availability

The datasets generated for this study are available on request to the corresponding authors.

## Supplementary Materials

The following supporting information can be downloaded at: https://www.mdpi.com/article/10.3390/s23125478/s1. Video S1: Animation of the heating and melting of sample 1 and sample 2 together with changing of the linear concentrations of Au and Pt atoms and temperature dependence of Lindemann index. Video shows the general view of the nanoparticles, for clearer observation of the thermal degradation of the surface and cross-section of the nanoparticles, for visibility of thermal degradation of the inner triple layered core-shell structure. Video S2: Animation of the heating and melting of five samples of Au@Pt@Au NP with different radius together with related temperature dependencies of Lindemann index.
